# Three Loci Affecting Variance of Body Mass Index in African Americans and Sub‐Saharan Africans

**DOI:** 10.1002/gepi.70009

**Published:** 2025-05-05

**Authors:** Daniel Shriner, Amy R. Bentley, Ayo P. Doumatey, Jie Zhou, Guanjie Chen, Charles N. Rotimi, Adebowale A. Adeyemo

**Affiliations:** ^1^ Center for Research on Genomics and Global Health National Human Genome Research Institute Bethesda Maryland USA

## Abstract

Conventional genome‐wide association studies (GWAS) are designed to assess the effect of a genetic locus on phenotypic mean by genotype. Such loci explain a proportion of phenotypic variance known as narrow‐sense heritability. In contrast, variance quantitative trait loci (vQTL) are associated with the phenotypic variance by genotype. These loci explain an additional proportion of phenotypic variance and contribute to broad‐sense heritability but not to narrow‐sense heritability. Here, a genome‐wide vQTL analysis in 22,805 African Americans yielded eight loci for body mass index (BMI). Of these loci, three were replicated in 6002 sub‐Saharan Africans. No locus reached genome‐wide significance using the standard additive model. Furthermore, no locus showed evidence for natural selection, haplotype effects, or gene × sex or gene × study interactions. Two loci showed evidence for an effect of locus‐specific ancestry resulting from admixture and for a gene × gene interaction. One locus showed evidence for interaction with diastolic blood pressure, consistent with this vQTL capturing an unmodeled gene × covariate interaction. These analyses demonstrate that relevant BMI loci can be detected by evaluating vQTL and that these loci contribute to the underexplored broad‐sense heritability for this trait.

## 1 Introduction

Narrow‐sense heritability is defined as the ratio of additive genetic variance to total phenotypic variance and is interrogated by conventional genome‐wide association studies assuming the standard additive genetic model. In contrast, broad‐sense heritability is defined as the ratio of total genetic variance to total phenotypic variance, capturing the contribution of non‐additive genetics to total phenotypic variance. One way to examine non‐additive genetics is to assess phenotypic variance by genotype by searching for variance quantitative trait loci (vQTL) (Paré et al. 2010; Rönnegård and Valdar 2011, 2012). There are several possible explanations for phenotypic variance differing by genotype, including unmodeled gene × gene and gene × covariate interactions (Cao et al. 2014; Ek et al. 2018). Testing for the presence of vQTL requires genotype data but does not require knowledge of, or data for, potential interactors (Struchalin et al. 2010).

Body mass index (BMI) is a complex quantitative trait. Comparison of estimates of narrow‐sense heritability to broad‐sense heritability suggests substantial contributions of non‐additive genetics to adult BMI (Robinson et al. 2017; Yang et al. 2011). Previous studies of BMI have reported gene × covariate interactions between genetic variants such as those in *FTO* and covariates such as alcohol intake, diet, and physical activity (Marderstein et al. 2021; Wang et al. 2019; Young et al. 2018). These studies were limited to British individuals with European ancestry.

African Americans are both genetically and environmentally diverse compared to British individuals of European ancestry, such that vQTL might not transfer across groups. Here, we extend the search for vQTL for BMI to African Americans. Whereas African Americans have approximately 80% sub‐Saharan African ancestry (Shriner et al. 2009), we attempt replication in an independent study of sub‐Saharan Africans. We describe evidence for three vQTL not previously reported in individuals of European ancestry, with all three loci mapping to regulatory variants. In contrast to previous findings in individuals of European ancestry, we find no evidence that *FTO* is a vQTL in individuals of African ancestry. Also, we investigate several possible explanations for the presence of the three vQTL. We detect varying contributions of admixture, gene × gene interactions, and gene × covariate interactions to the presence of the three vQTL.

## 2 Materials and Methods

### 2.1 Discovery Studies

The discovery studies comprised eight data sets of African Americans. The Atherosclerosis Risk in Communities (ARIC) Study is a prospective longitudinal study of cardiovascular disease in adults (The ARIC Investigators 1989). The Cleveland Family Study (CFS) is a prospective longitudinal study of sleep apnea in families (Redline et al. 1995). The Genetic Epidemiology Network of Arteriopathy (GENOA) is a prospective longitudinal study of hypertension in sibships (Daniels et al. 2004). The Howard University Family Study (HUFS) is a cross‐sectional study in families (Adeyemo et al. 2009). The Jackson Heart Study (JHS) is a prospective longitudinal study of cardiovascular disease in one community (Taylor et al. 2005). The Multi‐Ethnic Study of Atherosclerosis (MESA) is a prospective longitudinal study of cardiovascular disease in adults (Bild 2002). The Sea Islands Genetic Network (SIGNET) is a case‐control study of type 2 diabetes in one community (Garvey et al. 2003). The Women's Health Initiative (WHI) is a prospective longitudinal study of women's health in postmenopausal women (Assaf and Carleton 1994). Each study performed genotyping using the Affymetrix Genome‐Wide Human SNP Array 6.0. Using PLINK (version 1.90b2k), quality control was performed separately by study, filtering for individual call rate ≥ 90%, marker call rate ≥ 95%, and Hardy−Weinberg equilibrium *p *≥ 1 × 10^−10^. Monomorphic and strand‐ambiguous markers were removed. The bcftools (version 1.9) plugin fixref was used to fix reference allele mismatches with the human reference sequence (human_g1k_v37.fasta). The Python script checkVCF.py (version 1.4) was used to check input vcf files before imputation. Imputation was performed through the TOPMed Imputation Server using the r2 panel, filtering for rsq ≥ 0.3 and minor allele frequency ≥ 0.5%. The eight imputed data sets were then merged using bcftools.

### 2.2 Discovery Analysis

Phenotypic variables included BMI (kg/m^2^), age (years), and sex. Using GCTA (version 1.93.2beta), we estimated the genetic relatedness matrix and extracted the first principal component. Using the R package GMMAT (version 1.3.1), we obtained the residuals from the generalized linear mixed model regressing BMI on age, sex, study, and the first principal component as fixed effects and the genetic relatedness matrix as a random effect, using the identity link function. Using the R package stats, we performed the rank‐based Fligner−Killeen test of homogeneity of variance (Fligner and Killeen 1976) using the residuals and the most probable genotypes. The R implementation of the test uses median centering. The Fligner−Killeen test is robust to heterogeneity of phenotypic means by genotype under additive, dominant, or recessive models but is sensitive to the combined effects of skewness and excess kurtosis in the phenotypic distribution (Conover et al. 1981). To further investigate the validity and power of the Fligner−Killeen test, we simulated two groups of size 100,000 from different distributions (Supporting Information S1: Table S1). For each comparison, we generated 10,000 independent data sets and set the significance level to 0.05.

### 2.3 Replication Study

Replication data were drawn from the Africa America Diabetes Mellitus (AADM) study, comprised of participants from Ghana and Nigeria (West Africa) and Kenya (East Africa) (Adeyemo et al. 2019). To account for the fact that this study used several genotyping arrays, quality control and imputation were performed separately for each array as described under Discovery Studies. The five imputed data sets were merged using bcftools.

### 2.4 Replication Analysis

Phenotypic variables included BMI, age, sex, and genotyping array. Using GCTA, we estimated the genetic relatedness matrix and extracted the top two principal components. Using GMMAT, we obtained the residuals from the generalized linear mixed model regressing BMI on age, sex, genotyping array, and the top two principal components as fixed effects and the genetic relatedness matrix as a random effect, using the identity link function. Using R, we performed the rank‐based Fligner−Killeen test of homogeneity of variance using the residuals and the most probable genotypes.

### 2.5 Phenotypic Variance Explained

*P* values from the Fligner−Killeen test were converted into *t*‐statistics which were used to obtain unbiased estimates of the correlation coefficient *r* based on the formula $r=\frac{t}{\sqrt{t^{2}+n-2}}$, given a sample size n. An unbiased estimate of the proportion of phenotypic variance explained $r_{\mathrm{adj}}^{2}$ was then obtained from the formula $r_{\mathrm{adj}}^{2}=r^{2}-\frac{1-r^{2}}{n-2}$.

### 2.6 Annotation

Loci were manually annotated using Ensembl release 104 (Howe et al. 2021), HaploReg version 4.1 (Ward and Kellis 2016), and GTEx release 8.

### 2.7 Gene × Gene and Gene × Covariate Interactions

Gene × gene interactions were tested by regressing the residual BMI outcome on the most probable genotype at the first marker, the most probable genotype at the second marker, and their product. Gene × covariate interactions were tested by regressing the residual BMI outcome on the most probable genotype at a marker, the covariate, and their product. Current smoking and current drinking were analyzed as binary variables. Blood pressure was reported as the average of the second and third readings if three readings were taken, as the second reading if two readings were taken, or the first reading if one reading was taken.

### 2.8 Locus‐Specific Ancestry Inference

We used RFMix version 1.5.4 (Maples et al. 2013) to perform locus‐specific ancestry inference for the African Americans as previously described (Shriner et al. 2023). We inferred locus‐specific ancestry for each of the eight discovery studies separately and then merged the locus‐specific ancestry calls across the intersection of 527,318 markers that were genotyped in all eight studies.

## 3 Results

**Figure 1:**
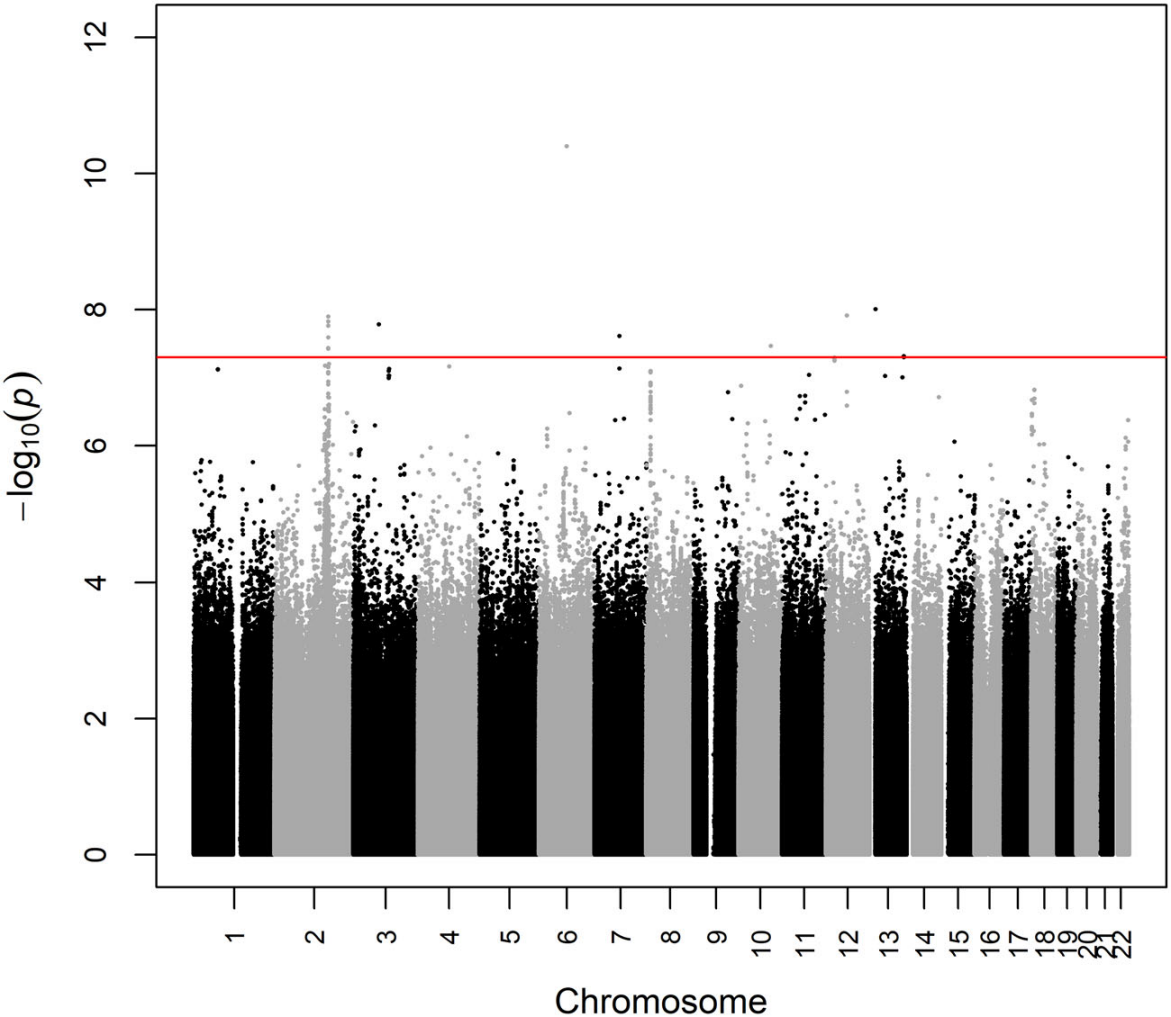
Manhattan plot from discovery analysis of vQTL for body mass index in African Americans.

We performed quality control and imputation separately for eight studies of African Americans (Supporting Information S1: Table S2). We merged the studies after imputation, yielding a discovery data set including 23,643 individuals and 16,503,295 markers. Similarly, we performed quality control and imputation for sub‐Saharan Africans from one study separately by genotyping array (Supporting Information S1: Table S3). We merged the data after imputation, yielding a replication data set including 6,051 individuals and 15,795,649 markers. BMI was regressed on age, sex, study (for African Americans), genotyping array (for sub‐Saharan Africans), and top principal components (one for African Americans and two for sub‐Saharan Africans, Supporting Information S2: Figure S1 and Figure S2, respectively) as fixed effects and the genetic relatedness matrix as a random effect (to account for both known and cryptic relatedness) using a generalized linear mixed model. Residuals were tested against genotype using the rank‐based Fligner−Killeen test of homogeneity of variances. Accounting for missing phenotype and covariate data, the discovery analysis comprised 22,805 African American individuals and the replication analysis comprised 6,002 African individuals. Of 15 markers in eight loci that reached genome‐wide significance in the discovery analysis (Figure 1), six markers in three loci replicated (Table 1). All six markers were either genotyped or had high imputation quality (Supporting Information S1: Table S4). Phenotypic variance explained by each vQTL ranged from 0.13% to 0.19% in the discovery data set and from 0.06% to 0.08% in the replication data set (Table 1).

**Table 1:** Discovery and replication results from genome‐wide vQTL analysis of body mass index.

						Discovery								Replication				
Chr	SNP	RefSeq ID	Position (bp)^a^	Minor allele	Major allele	*p* value	*r* ^2^_adj_	MAF^b^	HWE. pvalue	Additive. pvalue	Regulatory feature	Consequence	Gene	*p* value	*r* ^2^_adj_	MAF^2^	HWE. pvalue	Additive. pvalue
2	chr2:164648055:G:T	rs12692735	164,648,055	G	T	3.68E−08	1.28E−03	0.314	0.9634	3.52E−06		Intergenic		0.03410188	5.82E−04	0.2422	0.4193	0.4086
2	chr2:164655284:C:G	rs10179126	164,655,284	C	G	2.56E−08	1.32E−03	0.3133	1	7.54E−06	ENSR00001040126	Regulatory region variant	*COBLL1*	0.02385151	6.84E−04	0.2416	0.3604	0.3811
2	chr2:164668241:C:G	rs36105243	164,668,241	C	G	1.73E−08	1.35E−03	0.3162	0.9513	3.83E−06		Intronic	*COBLL1*	0.01431195	8.33E−04	0.2415	0.3791	0.3381
2	chr2:164669828:T:C	rs7592412	164,669,828	T	C	1.49E−08	1.36E−03	0.3158	0.9878	3.48E−06		Intronic	*COBLL1*	0.01687543	7.85E−04	0.2413	0.3981	0.3623
2	chr2:164687940:G:A	rs6738627	164,687,940	G	A	1.26E−08	1.38E−03	0.317	0.7033	8.99E−06		Intronic	*COBLL1*	0.0650255	4.01E−04	0.2465	0.2521	0.4588
2	chr2:164688863:C:CAA		164,688,863	C	CAA	3.77E−08	1.28E−03	0.2705	0.3246	4.82E−05		Intronic	*COBLL1*	0.2937594	1.71E−05	0.1994	0.03569	0.5618
3	chr3:78354830:C:T	rs190121444	78,354,830	T	C	1.65E−08	1.35E−03	0.00897	0.6999	0.8461		Intergenic		0.3790635	−3.87E−05	0.00798	0.312	0.5819
6	chr6:85227974:C:T	rs147864906	85,227,974	T	C	3.95E−11	1.87E−03	0.01174	0.5488	0.05679		Intergenic		0.0311676	6.07E−04	0.01206	0.5854	0.6366
7	chr7:76211003:A:G	rs7798565	76,211,003	G	A	2.46E−08	1.32E−03	0.2733	0.4935	0.008402		Intronic	*SRRM3*	0.02781348	6.40E−04	0.2925	0.8762	0.6564
10	chr10:99498312:C:G	rs56866375	99,498,312	G	C	3.40E−08	1.29E−03	0.02648	0.3596	0.3969		Intergenic		0.6434473	−1.31E−04	0.03743	0.5892	0.6563
12	chr12:64004860:C:T	rs115517761	64,004,860	T	C	1.22E−08	1.38E−03	0.02398	0.2467	0.01904	ENSR00001191801	Regulatory region variant	*SRGAP1*	0.8217791	−1.58E−04	0.03561	0.02263	0.3227
13	chr13:19105986:G:A	rs534466881	19,105,986	A	G	9.88E−09	1.40E−03	0.01284	0.793	0.2327		Intronic	lncRNA	0.833989	−1.59E−04	0.0133	0.6285	0.5737
13	chr13:105972955:T:G	rs114804599	105,972,955	G	T	4.93E−08	1.26E−03	0.00954	1	0.9352	ENSR00000489922	Regulatory region variant	lncRNA	0.3690827	−3.22E−05	0.01322	1	0.76
13	chr13:105973696:A:C	rs116107204	105,973,696	C	A	4.82E−08	1.26E−03	0.00967	1	0.9863		Intronic	lncRNA	0.3679336	−3.15E−05	0.0133	1	0.735
13	chr13:105973780:A:AT		105,973,780	AT	A	4.98E−08	1.26E−03	0.00956	1	0.9003				0.3618914	−2.81E−05	0.01322	1	0.776

^a^ Based on GRCh38.

^b^ Minor allele frequency.

**Figure 2:**
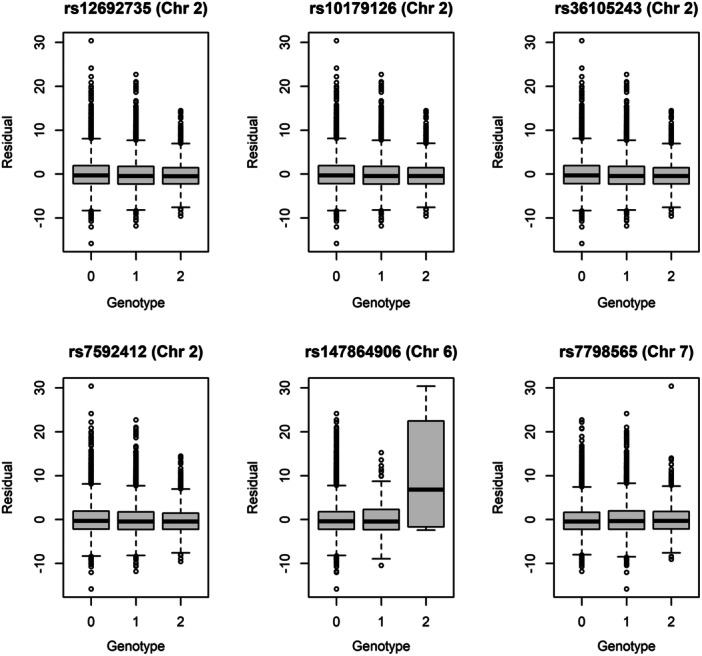
Boxplots of outcome versus genotype at the replicated loci. The plots show the distribution of the residual body mass index outcome as a function of the number of copies of the minor allele. The central value is the median, the box shows the first and third quartiles, and the whiskers represent ±1.58 times the interquartile range divided by sqrt(*n*), which is approximately the 95% confidence interval.

At the chromosome 2 locus, variance decreased as the number of minor alleles increased at each of the four markers (Figure 2). Three of four markers are intronic in *COBLL* and the fourth marker is intergenic. The four markers are in strong pairwise linkage disequilibrium (all pairwise *r* ^2^ ≥ 0.982) in the discovery data. At the chromosome 6 locus, variance increased as the number of minor alleles increased (Figure 2). The minor allele frequency at the SNP rs147864906 was 1.2%, and the genotype with two minor alleles at rs147864906 had a count of four. Due to this sparsity, we repeated the test in individuals with zero or one minor alleles and the significance was reduced from 3.95 × 10^−11^ to 2.24 × 10^−3^. Even though the Fligner−Killeen test is robust to differences in group means, the variance among minor allele homozygotes was poorly estimated with such a small sample size. Therefore, we advise caution in interpreting results at this marker. At the chromosome 7 locus, variance was largest for heterozygotes (Figure 2).

A vQTL can be a locus under natural selection. For example, if extreme phenotypic values are associated with reduced fitness, then the underlying genotype distribution might not be in Hardy‐Weinberg equilibrium. All six markers showed Hardy−Weinberg equilibrium (minimum *p *= 0.4935 in the discovery data and 0.3604 in the replication data), providing evidence against this scenario (Table 1).

At the vQTL on chromosome 2, the haplotype containing the major (and ancestral) allele at each marker had a frequency of 68.1% in the discovery data. Similarly, the haplotype containing the minor (and derived) allele at each marker had a frequency of 31.5%. Nine haplotypes collectively accounted for the remaining 0.4%. The test of homogeneity of variances based on the two most frequent haplotypes was genome‐wide significant (*p* = 1.61 × 10^−8^), comparable to the genotype‐based results for rs36105243 (*p* = 1.73 × 10^−8^) and rs7592412 (*p* = 1.49 × 10^−8^). A difference test of all haplotypes versus the two most frequent haplotypes was not significant (*p *= 0.465), indicating that the cumulative effect of the nine rare haplotypes was negligible (Supporting Information S1: Table S5). These results provide evidence against the explanation that the presence of a vQTL at this locus reflected multiple causal haplotypes.

**Figure 3:**
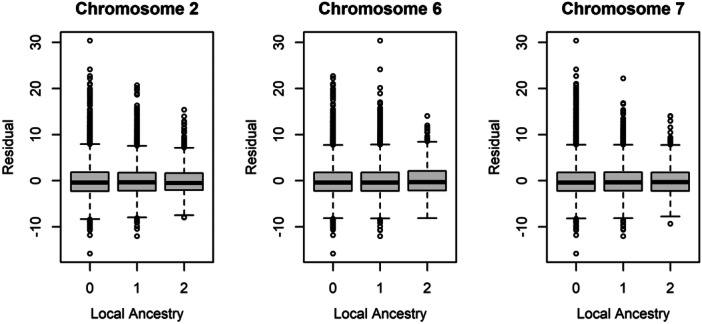
Boxplots of outcome versus locus‐specific ancestry at the replicated loci. The plots show the distribution of the residual body mass index outcome as a function of the number of chromosomes from the European ancestral background. The central value is the median, the box shows the first and third quartiles, and the whiskers represent ±1.58 times the interquartile range divided by sqrt(*n*).

**Figure 4:**
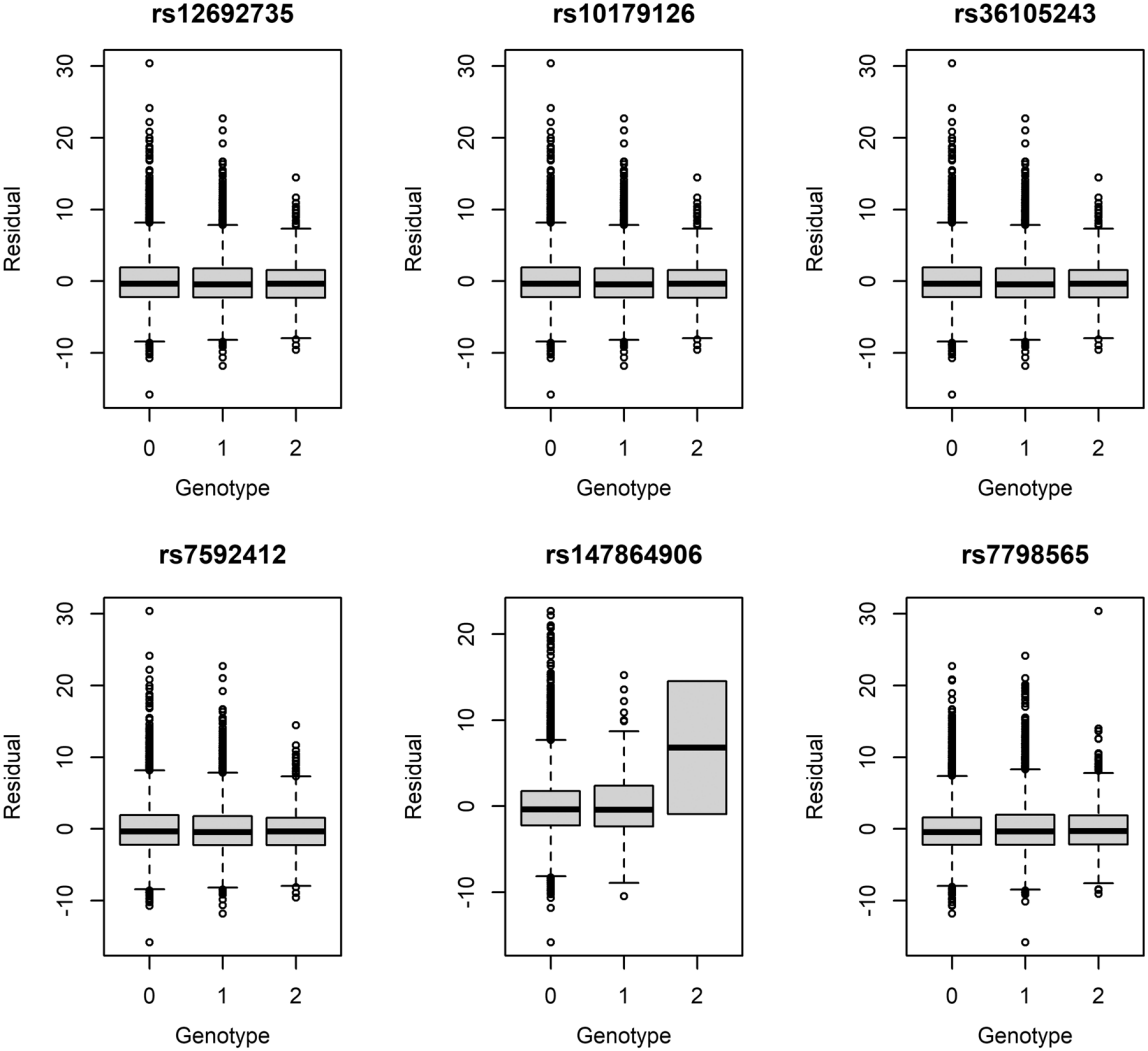
Boxplots of outcome versus genotype conditional on locus‐specific ancestry at the replicated loci. The plots show the distribution of the residual body mass index outcome as a function of the number of copies of the minor allele conditional on two chromosomes from the African ancestral background. The central value is the median, the box shows the first and third quartiles, and the whiskers represent ±1.58 times the interquartile range divided by sqrt(*n*).

A vQTL can indicate the presence of population stratification. Given a discovery data set of admixed African Americans, we tested the residual BMI outcome against locus‐specific ancestry. We observed significant effects of locus‐specific ancestry at the loci on chromosomes 2 and 7 (*p* = 1.30 × 10^−4^ and 0.0449, respectively), but not at the locus on chromosome 6 (*p *= 0.744). At the loci on chromosomes 2 and 7, variance decreased as the amount of African ancestry decreased (Figure 3). Additionally, at chromosome 2, all four minor alleles had a frequency of 22.5% in the African background but were the major alleles in the European background, with frequencies ranging from 58.5% to 59.6% (*F*_ST_ = 0.237−0.248). At chromosome 7, the minor allele varied from 31.3% in the African background to 16.1% in the European background (*F*_ST_ = 0.061). To remove locus‐specific ancestry effects induced by admixture, we tested genotypes within the stratum of locus‐specific ancestry defined by homozygous African ancestry (Figure 4). At the chromosome 2 locus, all four markers still yielded evidence for the presence of a vQTL (*p* from 1.27 × 10^−3^ to 1.83 × 10^−3^). The phenotypic variance explained was approximately 0.06%, indistinguishable from the amount explained in the African replication data set. Based on these results, we estimated that admixture explained approximately 53% of the signal at the chromosome 2 locus.

To eliminate confounding due to variation in parental ancestry when the confounding locus acts in an additive manner, it is sufficient to control for genome‐wide ancestry (Redden et al. 2006). To eliminate confounding due to variation in parental ancestry when the confounding locus acts in a non‐additive manner, it is sufficient to control for the proportion of the genome that is homozygous for African ancestry, in addition to controlling for genome‐wide ancestry (Redden et al. 2006). Inclusion in the null model of the first principal component in the African American data controls for genome‐wide ancestry, and therefore for confounding when the confounding locus acts in an additive manner. To test for the presence of confounding by variation in parental ancestry when the confounding locus acts in a non‐additive manner, we compared the residual BMI outcome and the proportion of the genome homozygous for African ancestry. After inverse‐normal transforming both variables, we observed no evidence that the residual BMI outcome was associated with variation in parental ancestry (Wilcoxon signed‐rank test, *p* = 0.697). This result indicates that the detection of vQTL is not a false positive result due to confounding from population stratification in the form of variation in parental ancestry.

Another possible explanation for a vQTL is a locus with an unmodeled gene × gene interaction. To test this explanation, we note that if one interacting locus is a vQTL, then the other interacting locus should also be a vQTL. Therefore, we performed pairwise comparisons for the six replicated markers. For all 15 pairwise comparisons, we observed point‐wise significance for the interaction term only for the chromosome 6 × chromosome 7 test (Supporting Information S1: Table S6), with the interaction effect explaining 9% of the signal at the chromosome 6 vQTL and 13% of the signal at the chromosome 7 vQTL. Similarly, a vQTL can be a locus with unmodeled gene × sex or gene × study interactions. However, none of the six markers showed evidence for these types of interactions (all *p* > 0.05).

A vQTL can also be a locus with an unmodeled gene × covariate (or environment) interaction. To test this explanation, we tested current smoking and current alcohol drinking (beer, wine, or liquor) as potential interacting variables with available questionnaire data. Although both variables had significant main effects (*p* = 1.46 × 10^−41^ and 2.07 × 10^−13^, respectively), we observed no significant interactions with any of the six replicated markers (Supporting Information S1: Table S7 and Table S8). Additionally, we investigated both systolic blood pressure (SBP) and diastolic blood pressure (DBP) (Julius et al. 2000). Both SBP and DBP had strong associations with the residual BMI outcome (*p* = 2.08 × 10^−85^ and 1.82 × 10^−71^, respectively). None of the six markers had significant interaction effects with SBP (Supporting Information S1: Table S9). The marker at the chromosome 7 locus, rs7798565, had a point‐wise significant interaction effect with DBP (Supporting Information S1: Table S10), consistent with an unmodeled gene × DBP interaction explaining 20% of the signal at this vQTL.

## 4 Discussion

We have identified three novel associations between genetic loci and BMI based on heterogeneity in variance by genotype. Previous analysis of BMI in the subsample of British individuals with European ancestry in the UK Biobank yielded evidence for gene × covariate interactions between genetic variants in *FTO* and covariates such as alcohol intake and physical activity (Marderstein et al. 2021; Wang et al. 2019; Young et al. 2018). In contrast, no marker at *FTO* reached genome‐wide significance as a vQTL in African Americans. Collectively, these findings indicate key differences in genotype−phenotype relationships and possibly the genetic architecture of BMI between these two ancestrally diverse groups.

The locus on chromosome 2 is a splicing QTL for *COBLL* in visceral adipose tissue (*p* = 1.10 × 10^−14^) and an expression QTL (eQTL) for *SLC38A11* in multiple tissues including naïve adipose (*p* = 1.77 × 10^−8^). COBLL1 possesses an actin‐binding domain and is involved in cell morphology, cell growth, and migration (Takayama et al. 2018). SLC38A11 is a putative sodium‐coupled neutral amino acid transporter. Of the four markers at this locus, rs10179126 maps to a predicted TFCP2 binding site with differential binding by allele, and rs36105243 maps to predicted E2A, TBX5, and ZEB1 binding sites with differential binding by allele. Additionally, rs36105243 maps to an enhancer in blood, and rs7592412 maps to an enhancer in fat, skin, and mesenchymal stem cells. At the locus on chromosome 6, rs147864906 is intergenic and maps to a predicted CEBPB binding site with differential binding by allele located 222 kb upstream of *NT5E* (also known as *CD73*). At the locus on chromosome 7, rs7798565 is intronic in *SRRM3* and maps to a predicted ZBTB33 binding site with differential binding by allele. The locus is an eQTL for *STYXL1* in multiple tissues, including subcutaneous adipose tissue (*p* = 9.59 × 10^−32^).

The chromosome 2 locus has been previously associated with type 2 diabetes (Morris et al. 2012), triglycerides (Willer et al. 2013), waist‐hip ratio (Shungin et al. 2015), and circulating leptin (Kilpeläinen et al. 2016). No associations have been reported for the markers at the other two loci. In our discovery data set, no marker had genome‐wide significant association with BMI under the standard additive genetic model, although five of the six markers in the three vQTL yielded point‐wise significance and the sixth was just above that threshold (*p* = 0.06).

There are some notable strengths of vQTL analysis. One major advantage of vQTL analysis over modeling of gene × covariate interactions arises from the wide variety of potential environmental interactors, including those that are not captured or captured well by questionnaires or other standard measurements. Two, vQTL analysis can detect loci that contribute non‐additive genetics to phenotypic variance and thus help to explain broad‐sense heritability. In turn, the utility of polygenic risk scores could be improved by the incorporation of additional loci that explain a component of phenotypic variance in addition to the component attributed to additive genetics.

Our study has some notable limitations. One, the discovery analysis in African Americans yielded eight associations, of which three replicated in sub‐Saharan Africans. Given the admixed nature of African Americans, it is possible that one or more of the five associations that did not replicate in sub‐Saharan Africans might replicate in Europeans. However, evidence from vQTL studies from the UK Biobank (Marderstein et al. 2021; Wang et al. 2019; Young et al. 2018) do not support this possibility. Two, the environmental factors we tested were by no means an attempt to be exhaustive, so it is possible that other gene × covariate interactions could explain more of the vQTL signals.

In summary, we detected three vQTL for BMI in African Americans and sub‐Saharan Africans. One vQTL on chromosome 2 mapped to four tightly linked variants including two regulatory variants with potentially pleiotropic effects on two genes in multiple tissues. Two vQTL on chromosomes 6 and 7 both mapped to single regulatory variants. Compared to findings from European populations, these small numbers of variants per locus reflect more precise delineation of risk loci resulting from weak linkage disequilibrium in populations of African ancestry. We found that natural selection, haplotype effects, gene × sex interactions, and gene × study interactions are all unlikely to explain heterogeneity in variance by genotype at any of the three vQTL. In contrast, we found that locus‐specific ancestry effects resulting from admixture, gene × gene interactions, and gene × covariate interactions partially explained heterogeneous variance by genotype. In the context of gene × covariate interactions, differences in environmental exposures, especially when samples reflect diverse lifestyles, cultures, and geography, should be considered.

## Author Contributions

Daniel Shriner designed the study, analyzed the data, interpreted the results, and drafted the manuscript. Amy R. Bentley interpreted the results and revised the manuscript. Ayo P. Doumatey, Jie Zhou, and Guanjie Chen generated and managed the data. Charles N. Rotimi and Adebowale A. Adeyemo designed the study, interpreted the results, and revised the manuscript. All authors read and approved the final version of the manuscript.

## Ethics Statement

Ethical approval was obtained from the National Institutes of Health and from the ethical committees in each study site.

## Consent

All participants gave written informed consent.

## Conflicts of Interest

The authors declare no conflicts of interest.

## Code Availability

GCTA is freely available from https://cnsgenomics.com/software/gcta/#Download.

PLINK is freely available from https://www.cog-genomics.org/plink/.

Bcftools is freely available from http://www.htslib.org/download/.

The R package GMMAT is freely available from https://cran.r-project.org/web/packages/GMMAT/index.html.

The R package stats is included in the standard library freely available at http://cran.r-project.org.

RFMix is freely available at https://www.dropbox.com/s/cmq4saduh9gozi9/RFMix_v1.5.4.zip.

## Supporting information

Supplementary Materials.

Supplementary Tables.

## Data Availability

The data sets used for the analyses in this manuscript were obtained from dbGaP through dbGaP accession study numbers phs000280 (ARIC), phs000284 (CFS), phs001238 (GENOA), phs000286 (JHS), phs000209 (MESA), phs000433 (SIGNET), and phs000200 (WHI). The HUFS and AADM data sets used and/or analyzed in the current study are available from C. N. Rotimi upon reasonable request. Genome‐wide summary statistics from the discovery study are freely available at https://www.genome.gov/sites/default/files/media/files/2024-03/aa_bmi_vqtl_txt.gz.
