# Supplementary material for: Three Loci Affecting Variance of Body Mass Index in African Americans and Sub‐Saharan Africans
Source: Genet Epidemiol. 2025 May 5;49(4):e70009. doi: 10.1002/gepi.70009 (PMC12051743; doi:10.1002/gepi.70009)
Supplement: Supplementary file 1 — Supplementary Materials. [file GEPI-49-0-s001.docx]

**Three Loci Affecting Variance of Body Mass Index in African Americans and sub-Saharan Africans**

Daniel Shriner, Amy R. Bentley, Ayo P. Doumatey, Jie Zhou, Guanjie Chen, Charles N. Rotimi, Adebowale A. Adeyemo

Center for Research on Genomics and Global Health, National Human Genome Research Institute, Bethesda, Maryland, USA

Supplementary Figure S1 p. 2

Supplementary Figure S2 p. 3

References p. 4


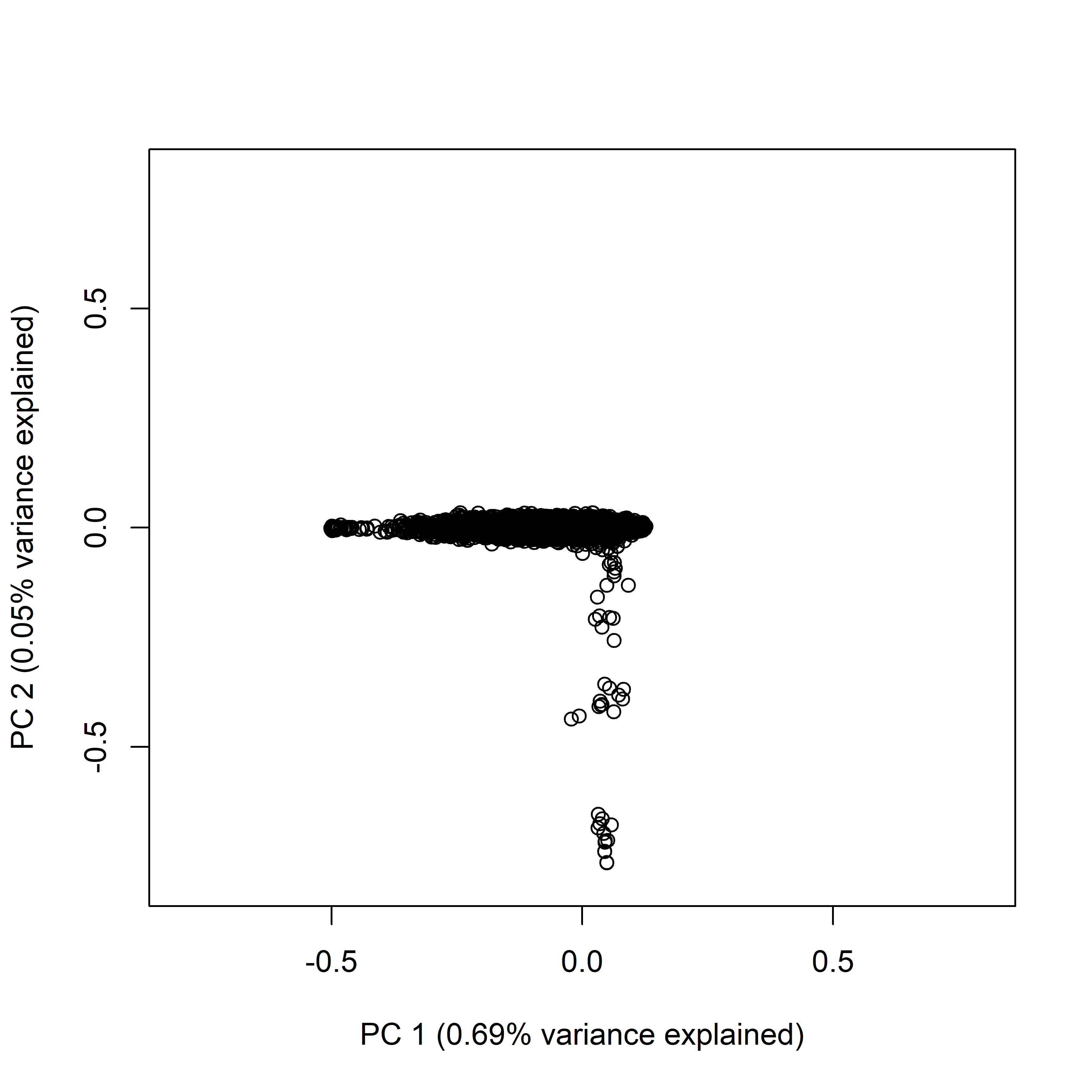


Supplementary Figure S1. Scatter plot of top two principal components of the genetic relatedness matrix in the discovery set of African Americans. The first principal component reflects two-way admixture. The second principal component reflects between- and within-study cryptic and known relatives (mostly from one family).


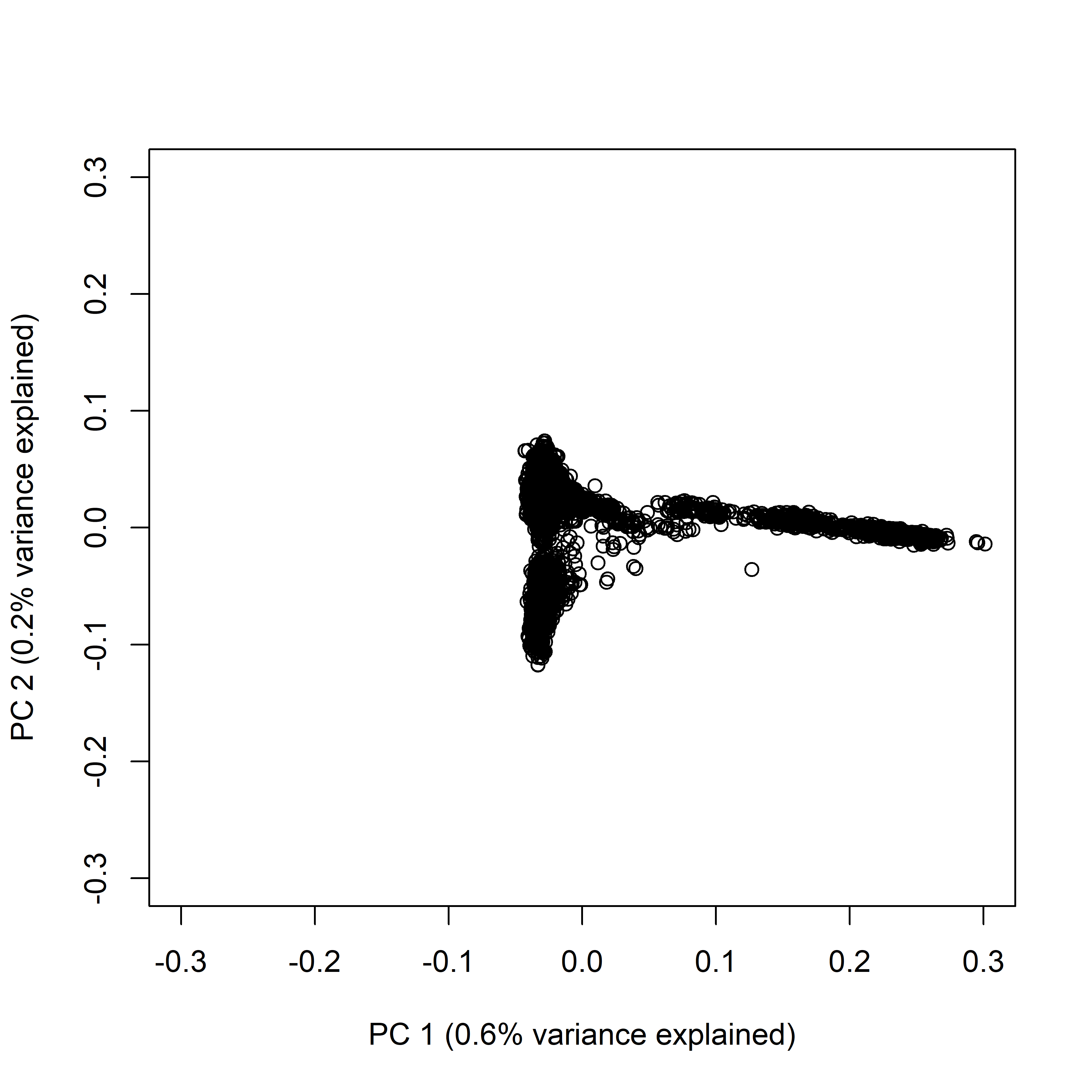


Supplementary Figure S2. Scatter plot of top two principal components of the genetic relatedness matrix in the replication set of sub-Saharan Africans. The first principal component separates Kenya from Nigeria and Ghana. The second principal component separates Nigeria and Ghana. Subsequent principal components reflect known and cryptic relatives (Liu, Shriner, Hansen, Rotimi, Mullikin, & Program, 2020).

References

Liu, Z., Shriner, D., Hansen, N. F., Rotimi, C. N., Mullikin, J. C., & Program, N. C. S. (2020). Admixture mapping identifies genetic regions associated with blood pressure phenotypes in African Americans. *PLoS ONE, 15*, e0232048. doi:10.1371/journal.pone.0232048
